# Development and validation of a guide for the continuity of care in perioperative medication management

**DOI:** 10.1186/s10195-018-0490-2

**Published:** 2018-08-27

**Authors:** Carmen Matoses-Chirivella, Andrés Navarro-Ruíz, Blanca Lumbreras

**Affiliations:** 1 Department of Pharmacy Services, University Hospital of Elche, Camino de la Almazara 11, 03203 Elche, Spain; 2 Department of Public Health, History of Science and Gynecology, Miguel Hernández University, Alicante, Spain; 3 CIBER en Epidemiología y Salud Pública (CIBERESP), Madrid, Spain

**Keywords:** Perioperative medication management, Guide, Concordance, Pharmacist

## Abstract

**Background:**

Increased longevity and the prevalence of associated pathologies is leading to more hospital admissions involving chronic patients with multiple pathological problems. In orthopedic surgical patients, it is very important to individually evaluate the risk/benefit of maintaining or suppressing chronic medications. For certain medications, there are consensus recommendations, but for others, the available information may be limited or controversial.

**Objective:**

To develop and validate a new guide for the continuity of care in perioperative medication management in older orthopedic surgical patients.

**Materials and methods:**

An expert pharmacist developed the guide by systematically reviewing each medication category according to the Anatomical Therapeutic Chemical (ATC) classification system. The Pharmacy and Therapeutics Committee at the Hospital General Universitario de Elche reviewed the guide. After a training course on the guide for pharmacists, the guide was validated by studying the interobserver variability between pharmacists as well as between each pharmacist and the expert pharmacist. Cohen’s kappa index (*κ*) was applied to determine interrater reliability.

**Results:**

The guide includes 51 therapeutic groups. Each ATC pharmacological subgroup is structured according to the benefits and risks of continuing therapy. When we compared each pharmacist’s recommendations with those of the expert pharmacist, the kappa value was found to be 0.8 [95% CI (0.7, 0.9)], indicating almost perfect concordance (overall percentage agreement 89.3%).

**Conclusions:**

We developed a guide for the continuity of care in perioperative medication management to improve the rationalization of medicines in the perioperative environment. After the pharmacists had been trained, the guide was validated by demonstrating a high level of concordance among the pharmacists’ recommendations. Formal training seems to be essential to ensure consistency in medical decisions.

**Level of evidence:**

IV (Oxford Centre for Evidence-Based Medicine. http://www.cebm.net/index.aspx?o=5653).

## Introduction

Chronic medication management is essential in order to provide optimal care for the older orthopedic surgical patient. The purpose of the study reported here was to provide guidance to health care professionals on medication management during the perioperative period.

In 2015, global life expectancy at birth was 76.8 years in the World Health Organization (WHO) European Region [1]. The prevalence of comorbidities in the elderly is high, with 80% of this population having three or more chronic conditions [2]. Increases in longevity and the prevalence of associated pathologies are reflected in the fact that most hospital admissions involve chronic patients with multiple pathological problems [3, 4]. The rising population aged more than 64 years has also resulted in a higher than expected prevalence and incidence of bone fractures [5].

In recent years, a significant proportion of medication errors have occurred during transitions between levels of care, especially during admission and discharge [6]. In 2005, the WHO launched the Action on Patient Safety initiative, also known as the High 5s project, to address issues related to the safety of patients around the world [7]. This initiative includes, among others, a protocol to assure medication accuracy at transitions in care or medication reconciliation. In hospitals that implemented this protocol, the morbidity and mortality associated with medication errors were reduced by 32% [8].

Kennedy et al. [9] carried out a prospective survey to identify drug usage/withdrawal in surgical patients and its relationship to the relative risk for postoperative surgical complications. The researchers concluded that at least 50% of patients who were undergoing surgery took medications on a regular basis that were not related to their surgery. Moreover, they stated that withdrawing regular medicines may significantly increase the risk of surgery and further complicate the outcome.

Clinicians must often decide whether chronic medications should be continued during the perioperative period. Unfortunately, there is a lack of medical evidence in this regard, which is reflected in considerable variability in perioperative management recommendations. Kroenke et al. [10] assessed opinions regarding the preoperative discontinuation or modification of selected medications by mailing a questionnaire to all 150 anesthesiology program directors in the United States. The responses highlighted great variation in practice medication management, reflecting a lack of firm evidence favoring any one approach.

Among orthopedic surgical patients, it is very important to individually evaluate the risk/benefit of maintaining or suppressing chronic medications, which will depend partly on the drug and the type of surgical intervention, but most importantly on the clinical status of the patient [11]. However, given the lack of sound evidence on this topic, clinicians base their decisions on expert opinions, isolated clinical cases, or theoretical considerations based on experience with similar drugs [12].

Hence, it is necessary to gather together and evaluate the available recommendations for maintaining or suppressing chronic medications during the perioperative period [13, 14], and then to use this information to produce a guide for the continuity of care in perioperative medication management. Such a guide could help hospital pharmacists to ensure the continuity of chronic pharmacotherapeutic treatment, thereby avoiding unnecessary interruptions and searches for therapeutic alternatives. However, this guide would not be a substitute for clinical judgment and experience.

The aim of the present study was therefore to develop (by reviewing the available evidence) and to validate a new guide for the continuity of care in perioperative medication management, which could aid pharmacists and surgeons who need to manage chronic medications in older adults during the perioperative period.

## Materials and methods

### Study design

**Fig. 1:**
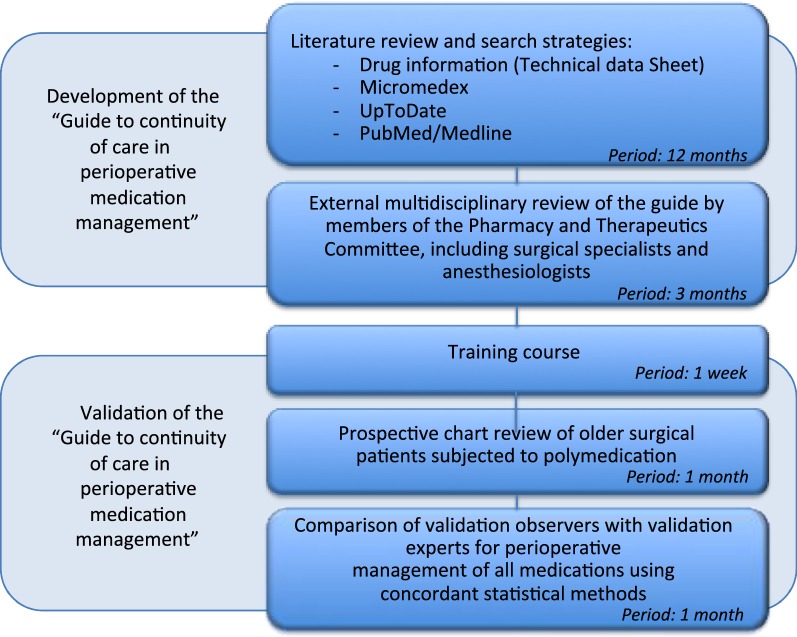
Study design

The development of the guide for the continuity of care in perioperative medication management was based on a literature search and an external review by an expert committee. The guide was validated through a prospective, noninterventional cohort study. The flow of the study process is illustrated in Fig. 1.

#### Development of the guide

The guide was formulated by an expert pharmacist (CM) by systematically reviewing the available evidence for each medication class, based on the Anatomical Therapeutic Chemical (ATC) classification system developed by the European Pharmaceutical Market Research Association [15]. It includes the most consumed ATC pharmacological subgroups according to data for the year 2014 from the Ministry of Health, Social Services and Equality of Spain [16].

Recommendations were based on three concepts: the pharmacokinetics of the drug, the effect of withdrawing the medication on the primary disease, and the effect of the medicine on the perioperative risk, including potential interactions with anesthetic agents.

For the literature search, a consistent process was applied, based on:

1. Drug information (technical data sheet).
2. Micromedex^®^. Provides summaries and detailed monographs for drugs, diseases, alternative medicine, toxicological managements, reproductive risks, and emergency care. It includes the following drug information databases:
  DRUGDEX^®^ system. Dosage, pharmacokinetics, cautions, interactions, clinical applications, and comparative drug efficacy.
  MARTINDALE. Electronic version of the Martindale textbook published by the Royal Pharmaceutical Society of Great Britain. Offers extensive information on international drug products. Especially useful when searching for European drugs, and can be searched by brand name or generic name.
  Alternative medicine. Includes monographs on herbal, vitamin, mineral, and other dietary supplements, based on scientific evidence as well as historical and common uses.
3. UptoDate^®^. An evidence-based, physician-authored clinical decision support resource that clinicians trust to make the right point-of-care decisions. Muluk and Macpherson provide an overview of preoperative patient assessment as well as details about the perioperative management of specific medications [12].
4. PubMed^®^. Online database of biomedical journal citations and abstracts. The search strategy was similar to that applied by Lievanos Rojas in his thesis *Perioperative management of chronic medications in orthopaedic surgery. A systematic review of the literature* [17].

Finally, an external multidisciplinary review of the guide was performed by members of the Pharmacy and Therapeutics Committee at the Hospital General Universitario de Elche, including surgical specialists and physicians from the Department of Anesthesiology, who contributed their experience in clinical practice.

#### Validation of the guide

The guide was validated by performing an interobserver variability study.

### Participants

An expert pharmacist (CM) with 15 years of experience in the pharmacotherapeutic validation of medical orders was responsible for developing the guideline, and acted as the gold standard. She determined the correct action to perform regarding usual chronic treatments in the perioperative environment according to the clinical status of the patient.

The observers comprised eight pharmacists with different levels of professional experience who were working in the same hospital. There were three staff pharmacists, all of whom had clinical and pharmacological knowledge and a wide range of experience in the pharmacotherapeutic validation of medical orders; five resident pharmacists, two of whom were residents in their first year and thus had little knowledge of the practical application of drugs; and three other resident pharmacists in their second or third year of residency, who had more experience in validating the pharmacotherapeutic profiles of patients.

### Training course

The course was given by the expert (CM). The concepts covered in the session addressed the following five questions:

1. *Why was the guide created?* She explained that the purpose of the guide was to ensure the continuity of pharmacotherapeutic information, reduce variability in clinical practice, exceed the needs of the patient at all times during the perioperative period by improving safety, and improve the efficiency of the medication utilization process.
2. *How is the guide structured?* She presented a brief summary of the format of the guide, including its structure according to the ATC classification, as well as the benefits and risks of continuing or discontinuing medication in the perioperative environment.
3. *How are the chronic medications grouped according to perioperative recommendations?* She described simple concepts for the following situations:
  3.1. Drugs that can increase morbidity if they are discontinued abruptly. Their use should continue in the perioperative period, or the treatment can be adjusted if possible.
  3.2. Drugs that increase the risk of anesthetic medications or complications during surgery and which are not essential in the short term. These drugs should be suspended during the perioperative period.
  3.3. Drugs that do not belong to any of the previous groups. These may be suspended or continued according to clinical criteria.
4. *What basic pharmacological concepts do we need to know?* She gave participants a brief overview of the most relevant drug interactions as well as descriptions of metabolic processes and the elimination of drugs and their metabolites, and she discussed how these can be altered in the perioperative period.
5. *How should I act if I have any doubt?* She stressed the importance of agreeing with clinic staff (either the orthopedic surgeon responsible for the patient or another relevant medical specialist) on the action to be taken in the event of clinical instability of the patient, or if there is doubt about the typical chronic treatment.

### Source of patients

Patients admitted to an orthopedic surgery unit in a Spanish tertiary 450-bed hospital from August 1 to September 1, 2016, were included in the validation study. The number of chronic medications required for the study was calculated based on the sample size required to detect a kappa value that was significantly different from zero with 90% power. We aimed for a power of 90% in a two-tailed test for a kappa value of at least 0.6, where we estimated that the guidelines would have greater than 90% concordance with the views of the expert pharmacist. The calculated value was based on assessments of over 30 drugs [18]. Therefore, 140 drugs were analyzed in 20 patients (seven drugs per patient).

### Study procedure

Each observer (i.e., pharmacist) received a dossier containing drug therapy and clinical information about each of the 20 patients to whom the guide was to be applied. The information about the patients comprised the following: the patient’s ID number (1–20), age, sex, personal history, diagnosis-related drugs (DRGs), date of surgical intervention, and chronic treatment. The form included specific instructions that had to be marked with an X depending on whether the decision was made to continue (C) or suspend (S) treatment for the patient according to the guide for the continuity of care in perioperative medication management and the clinical information about the patient.

Patient treatments were reviewed blindly and independently by the eight pharmacists and compared with the gold standard (CM).

### Statistical analysis

Statistical analyses were carried out using the software SPSS for Windows 20.0 (IBM SPSS). Cohen’s kappa, with a confidence interval (CI) of 95%, was used to analyze the concordance between each observer and the expert and between the eight observers. The degree of concordance was expressed as a numerical value of* k*, which ranged from 0.0, indicating absolute discordance, to 1.0, indicating perfect concordance. A value of > 0.61 indicated that the agreement was good [19]. For each item in the scale, the percent agreement was calculated as the number of times that the raters agreed on a rating (continue/discontinue) divided by the total number of ratings.

## Results

### Development of the guidelines

Some of the information reviewed came from clinical trials, but most was based on the opinions of experts, isolated clinical cases, or theoretical considerations according to experience with similar drugs [12]. There are consensus recommendations for several medications, whereas information is limited or controversial for others. Therefore, it is very important to assess the risk/benefit ratio in each case, and it is possible that the final decision will not coincide with the general recommendations. For each drug, we selected several articles reviewing the full text of all relevants.

After reviewing the available information on the perioperative management of chronic medications in order to develop the guide, Table 1 was created. It divides the drugs into 12 main anatomical groups, 51 therapeutic groups, and a phytotherapy revision group.

**Table 1:** Perioperative management of medications

Class	Benefits in continuing therapy	Risks in continuing therapy	Considerations	Recommendation
*A: Alimentary tract and metabolism*				
A02B: Drugs for peptic ulcer and gastro-oesophageal reflux disease (GORD)	Prevents stress-related mucosal damage caused by surgery, decreases gastric volume and raises gastric fluid pH, reducing the risk of chemical pneumonitis from aspiration	PPIs increase the risk of* Clostridium difficile* infection	Essential prior to anesthesia	Continue as usual
A03A: Drugs for functional gastrointestinal disorders	Promotes gastric emptying	No known perioperative adverse effects	Baseline ECG required to document QT interval	Continue as usual
A06: Drugs for constipation		No known perioperative adverse effects		Continue as usual
A07E: Intestinal antiinflammatory agents		Increased bleeding risk due to antiplatelet effects		Discontinue
A10A: Insulins and analogues	Hyperglycemia increases the risk of perioperative infections	Induces hypoglycemia	Basal insulin therapy is necessary in all insulin-treated diabetic patients	Continue with adjustments
A10B: Blood-glucose-lowering drugs excl. insulins	Avoids perioperative hyperglycemia	Significant risk of hypoglycemia *Metformin:* contraindicated in conditions that increase the risk of renal hypoperfusion, lactate accumulation, and tissue hypoxia *Thiazolidinediones:* could precipitate congestive heart failure due to fluid retention and peripheral edema *DPP4 inhibitors and GLP*-*1 analogs*: alter gastrointestinal motility	Monitor blood glucose frequently	Should be taken until the day before the operation but discontinued the day of the operation
A12: Mineral supplements			Ensure that electrolyte balance is controlled	Discontinue
*B: Blood and blood-forming organs*				
B01: Antithrombotic agents		Increased bleeding risk		Refer to perioperative management of antiplatelet therapy guide
B03A: Iron preparations		Constipation risk in bedridden patients, which is increased with opioid therapy	Severe iron-deficiency anemia may require a blood transfusion	Discontinue
B03B: Vitamin B12 and folic acid				Discontinue
*C: Cardiovascular system*				
C01AA: Digitalis glycosides	Management of underlying atrial fibrillation or congestive heart failure		Narrow therapeutic window. Check digoxin levels	Continue as usual
C01BD: Antiarrhythmics, class III	Possibility of recurrence of arrhythmias if stopped	Bradycardia, electrolyte imbalances may exacerbate risk of QT prolongation with amiodarone	*Amiodarone:* long half-life	Should be continued until and including the day of the operation
C01DA: Organic nitrates	May precipitate chest pain if withheld	Hypotension		Should be continued until and including the day of the operation
C02CA: Alpha-adrenoreceptor antagonists		Risk of intraoperative floppy iris syndrome (IFIS) with cataract surgery. Hypotension		Continue
C03: Diuretics	Prevent decompensation of congestive heart failure (CHF)	Tissue damage and reduced kidney perfusion immediately postoperatively may contribute to the development of hyperkalemia, which may be additive with concurrent potassium-sparing diuretics		Should be taken until the day before the operation but discontinued the day of the operation, except in patients with CHF
C04: Peripheral vasodilators		Increased bleeding risk		Discontinue
C07: Beta-blocking agents	Reduce ischemia by decreasing myocardial oxygen demand due to increased catecholamine. Help to prevent or control arrhythmias	Bradycardia and hypotension Interacts with epinephrine	Rebound hypertension can occur if stopped abruptly Monitor blood pressure closely postoperatively Only some drugs are available as injections; it may be necessary to change to an alternative drug if an oral route is not available	Should be continued until and including the day of operation
C08: Calcium channel blockers	May precipitate chest pain if withheld	Rebound hypertension can occur if stopped abruptly	Monitor blood pressure closely postoperatively Only some drugs are available as injections; it may be necessary to change to an alternative drug if an oral route is not available	Should be continued until and including the day of the operation
C09: Agents acting on the renin–angiotensin system	Management of postoperative hypertension	Can decrease blood pressure at induction of anesthesia, and many drugs within this class have differing half-lives		Should be continued until the day before the operation but discontinued on the day of the operation. Last dose should be given 10 h before induction of anesthesia
C10: Lipid-modifying agents (non-statin)		*Niacin and fibric acid derivatives*: may increase risk of myopathy and rhabdomyolysis, especially when used in combination with statins *Bile acid sequestrants*: interfere with the absorption of other medications		Discontinue
C10AA: HMG-CoA reductase inhibitors	Provide cardiovascular protection	May increase the risk of myopathy and rhabdomyolysis		Continue as usual
*G: Genitourinary system and sex hormones*				
G03A: Hormonal contraceptives for systemic use		Increased risk of postoperative venous thromboembolism (VTE)		*Estrogen*-*containing oral contraceptives:* discontinue 4–6 weeks prior to surgery in patients with a high risk of VTE
G04BD: Drugs for urinary frequency and incontinence		Risk of arrhythmias		Continue as usual
*H: Systemic hormonal preparations, excl. sex hormones and insulins*				
H02AB: Glucocorticoids	Increased risk of Addisonian crisis if stopped	Impaired wound healing, increased superficial blood vessels, risk of fractures, infections, and gastrointestinal ulcer		Continue—add stress dosing if > 5 mg prednisone per day (or equivalent) in six months prior to surgery, or on chronic therapy
H03: Thyroid therapy		No known perioperative adverse events	Thyroid function should ideally be checked preoperatively to ensure euthyroid state	Should be continued until and including the day of the operation
*J: Antiinfectives for systemic use*				
J05A: Direct-acting antivirals	Incidence of postoperative bacterial complications and sepsis is increased in patients with lower CD4 cell counts if antiretroviral agents are discontinued		Most data regarding surgical morbidity and mortality in the HIV-infected patient predate the availability of effective antiretroviral therapy	Continue as usual
*L: Antineoplastic and immunomodulating agents*				
L01AB: Alkyl sulfonates	No studies suggest that stopping preoperatively reduces the incidence of infection or improves wound healing		The use of lower doses may permit safer use. Monitor renal function and blood count postoperatively	Continue as usual
L01XX: Other antineoplastic agents				Discontinue 3–4 days prior to surgery
L02BA: Anti-estrogens	If used for cancer treatment, disease progression may be of concern once treatment interrupted	Increased risk of venous thromboembolism		Discontinue 4–6 weeks prior to surgery in hip and knee surgery
L02BG: Aromatase inhibitors	If used for cancer treatment, disease progression may be of concern once treatment interrupted	Unknown perioperative effects		Continue as usual
L04AA: Selective immunosuppressants	Controlling rheumatoid response	Increased risk of myelosuppression and wound-healing complications postoperatively		*Abatacept:* discontinue prior to surgery at 2 months
L04AB: Tumor necrosis factor alpha (TNF-Α) inhibitors	Controlling rheumatoid response	Increased risk of myelosuppression and wound-healing complications postoperatively		Discontinue prior to surgery at a timing equal to 2–5 half-lives of the respective drug Mean half-life (days): infliximab (8–9, 5), etanercept (4–5), adalimumab (15–19)
*M: Musculoskeletal system*				
M03BX: Other centrally acting agents	Abrupt withdrawal of intrathecal baclofen may result in severe sequelae (hyperpyrexia, rebound/exaggerated spasticity, muscle rigidity, and rhabdomyolysis), leading to organ failure and fatality			Continue as usual
M04A: Antigout preparations	Surgery could precipitate acute gouty arthropathy			Continue as usual. Held on the morning of surgery
M05BA: Bisphosphonates		Esophagitis in bedridden patients		Discontinue
*N: Nervous system*				
N02A: Opioids	Abrupt withdrawal can cause yawning, abdominal cramps, nausea, vomiting, insomnia, anxiety, and salivation			Should be continued until and including the day of the operation without exception
N02B: Other analgesics and antipyretics	Aspirin (ASA) withdrawal linked to cardiovascular events	Continuation may cause perioperative hemorrhage		Continue ASA for secondary cardiovascular prevention Discontinue ASA for primary cardiovascular prevention
N03: Antiepileptics	Possibility of precipitating convulsions if stopped	*Phenytoin:* levels may fluctuate in response to perioperative situations *Carbamazepine:* interactions with medications administered in the perioperative period *Valproic acid:* thrombocytopenia	Check serum drug level	Should be continued until and including the day of the operation
N04: Antiparkinson drugs	Avoid symptoms of Parkinson’s disease (agitation, rigidity)	Metabolite of levodopa, dopamine can cause arrhythmias, hypotension or hypertension		Should be continued until and including the day of the operation
N05A: Antipsychotics	Withdrawal symptoms can occur if stopped abruptly plus severe agitation	Some agents are associated with QT prolongation, and occasionally cause hypotension or arrhythmias	A routine ECG should be performed on all patients preoperatively	Continue as usual
N05AN: Lithium	Decreases the release of neurotransmitters and may prolong the effect of neuromuscular blockers		Close monitoring of fluid and electrolytes is essential due to the narrow therapeutic index of lithium and the usual changes in electrolyte levels postoperatively	Should be continued until and including the day of the operation
N05B: Anxiolytics	Continue these agents to avoid withdrawal; however, the patient will likely have decreased anesthesia requirements	Risk of pharmacokinetic and pharmacodynamic interactions with drugs used in the perioperative setting	If a benzodiazepine becomes necessary, consider using short–medium half-lives	Continue if indicated
N06AA: Nonselective monoamine reuptake inhibitors	Withdrawal symptoms can occur if stopped abruptly	Arrhythmias with anesthetics		Continue as usual. Discontinue if arrythmia occurs
N06AB: Selective serotonin reuptake inhibitors	Withdrawal symptoms can occur if stopped abruptly	Bleeding risk, drug interactions		Continue as usual
N06AG: Monoamine oxidase A inhibitors	Risk of withdrawal symptoms	Interactions with medications used in the perioperative setting (hypertension)	Avoid administration of meperidine/dextromethorphan/ephedrine and monitor closely while on narcotics (potential for reactions consisting of rigidity, hallucinating, fever, confusion, coma, and death)	Discontinue
N06D: Antidementia drugs		Through their effects on acetylcholinesterase, these agents are likely to exaggerate muscle relaxation during anaesthesia produced by suxamethonium, hence prolonging neuromuscular blockade		The relevant pharmaceutical manufacturers recommend discontinuation of both of these agents preoperatively to avoid these effects
N07C: Antivertigo preparations				Continue as usual
*R: Respiratory system*				
R03: Drugs for obstructive airway diseases	*Inhaled bronchodilators:* may precipitate bronchospasm if withheld	*Theophylline:* risk of arrhythmias and neurotoxicity	*Theophylline*: narrow therapeutic range	Continue as usual *Theophylline:* discontinue evening before surgery
*S: Sensory organs*				
S01: Ophthalmologicals		No known perioperative adverse effects		Continue as usual
S02: Otologicals		No known perioperative adverse effects		Continue as usual
V: Various				
V03AE: Drugs for treatment of hyperkalemia and hyperphosphatemia		No known perioperative adverse effects		Continue as usual
V03AF: Detoxifying agents for antineoplastic treatment		No known perioperative adverse effects		Continue as usual
Phytotherapy	No evidence that phytotherapy improves surgical outcomes	*Ephedra:* increases the risk of heart attack and stroke *Garlic:* increases the risk of bleeding *Ginkgo:* increases the risk of bleeding *Ginseng:* lowers blood sugar and increases the risk of bleeding *Valerian:* increases the sedative effects of anesthetics and is associated with benzodiazepine-like withdrawal *Echinacea:* allergic reactions and immune stimulation		Should be discontinued at least one full week prior to the planned surgical procedure

*PPIs* proton pump inhibitors, *ECG* electrocardiogram

### Validation of the guidelines

#### Sample of patients selected for the observational concordance study

During the study period, 140 drugs were analyzed; those drugs were taken by 20 Caucasian patients (seven drugs/patient) admitted to the orthopedic surgery unit.

The demographic (age and sex) and clinical (number of comorbidities) characteristics and diagnosis-related groups (DRGs) of the 20 patients are described in Table 2.

**Table 2:** Demographic and clinical characteristics of the 20 patients

DRG	n (%)	Sex		Median age (years)	Median number of comorbidities per patient
		M	F		
209—Major joint and limb reattachment procedures for a lower extremity	9 (45.0)	2	7	78.56	3.44
211—Hip and femur procedures excluding a major joint, age > 17 years, without complications or comorbidities	2 (10.0)	2	0	73	4.5
218—Lower extremity and humerus procedures excluding hip, foot, and femur, age > 17 years, with complications or comorbidities	1 (5.0)	1	0	45	3
219—Lower extremity and humerus procedures excluding hip, foot, and femur, age > 17 years, without complications or comorbidities	2 (10.0)	1	1	47.5	3
251—Fracture, sprain, strain, and dislocation of forearm, hand, or foot, age > 17 years, without complications or comorbidities	1 (5.0)	0	1	38	4
807—Anterior and posterior spinal fusion combined, without complications	1 (5.0)	0	1	86	4
818—Hip replacement without complications	4 (20.0)	0	4	81.25	3.75
Total	20 (100.0)	6	14	70.45	3.60

*DRG* diagnosis-related group, *M* male, *F* female

In total, there were 72 major comorbidities in the 20 patients, with an average of 3.6 comorbidities per patient. The most frequently detected comorbidity was hypertension, in 13 patients (65%), followed by depression in six patients (30%), and congestive heart failure and diabetes mellitus in five patients each (25% each). There were also four cases of dyslipidemia (20%) and four of atrial fibrillation (20%). Only three cases of osteoporosis were detected (15%), three of acute myocardial infarction (15%), three of benign prostatic hyperplasia (3%), and three of dementia/Alzheimer’s disease (15%). Vertiginous syndrome was observed in two patients (10%), hiatal hernia in two patients (10%), and anemia in two patients (10%). Finally, other comorbidities such as stroke, ulcer, acquired immune deficiency syndrome, Parkinson’s disease, neoplasia, insomnia, hypothyroidism, gout, schizophrenia, epilepsy, Crohn’s disease, and asthma/chronic obstructive pulmonary disease were detected in 12 patients (60%) (data not shown).

#### Drugs reviewed in the study

The eight observers reviewed 140 drugs. The most prevalent therapeutic groups were group N (nervous system), 43 drugs (30.71%); group C (cardiovascular system), 37 medicines (26.43%); group A (alimentary tract and metabolism), 27 drugs (19.29%); and group B (blood and blood-forming organs), 14 drugs (10%) (Table 3).

**Table 3:** Absolute agreement among eight pharmaceutical observers following the application of the guide, listed according to ATC group

Medicine class	*n* (%)	Kappa value	Agreement
A02: Drugs for acid-related disorders	16 (11.4)	1	Almost perfect
A07: Antidiarrheals, intestinal anti-inflammatory/anti-infective agents	1 (0.7)	*p* < 0.01	Poor
A10: Drugs used in diabetes	8 (5.7)	0.69	Substantial
A11: Vitamins	1 (0.7)	1	Almost perfect
A12: Mineral supplements	2 (1.4)	0.75	Substantial
B01: Antithrombotic agents	10 (7.1)	0.16	Slight
B03: Antianemic preparations	4 (2.9)	0.81	Almost perfect
C01: Cardiac therapy	4 (2.9)	1	Almost perfect
C02: Antihypertensives	1 (0.7)	1	Almost perfect
C03: Diuretics	11 (7.9)	0.51	Moderate
C05: Vasoprotectives	1 (0.7)	1	Almost perfect
C07: Beta-blocking agents	1 (0.7)	1	Almost perfect
C08: Calcium channel blockers	3 (2.1)	1	Almost perfect
C09: Agents acting on the renin-angiotensin system	9 (6.4)	0.83	Almost perfect
C10: Lipid-modifying agents	6 (4.3)	0.33	Fair
D11: Other dermatological preparations	1 (0.7)	< 0.01	Poor
G03: Sex hormones and modulators of the genital system	1 (0.7)	1	Almost perfect
G04: Urologicals	2 (1.4)	0.55	Moderate
H02: Corticosteroids for systemic use	2 (1.4)	1	Almost perfect
H03: Thyroid therapy	3 (2.1)	1	Almost perfect
J05: Antivirals for systemic use	1 (0.7)	0.50	Moderate
L01: Antineoplastic agents	1 (0.7)	1	Almost perfect
L02: Endocrine therapy	1 (0.7)	1	Almost perfect
L04: Immunosuppressants	1 (0.7)	1	Almost perfect
M01: Anti-inflammatory and antirheumatic products	1 (0.7)	1	Almost perfect
M04: Antigout preparations	1 (0.7)	1	Almost perfect
N02: Analgesics	10 (7.1)	0.60	Moderate
N03: Antiepileptics	5 (3.6)	1	Almost perfect
N04: Antiparkinson drugs	2 (1.4)	0.46	Moderate
N05: Psycholeptics	14 (10.0)	0.93	Almost perfect
N06: Psychoanaleptics	11 (7.9)	0.76	Substantial
N07: Other nervous system drugs	1 (0.7)	0.50	Moderate
R03: Drugs for obstructive airway diseases	1 (0.7)	1	Almost perfect
S01: Ophthalmologicals	1 (0.7)	1	Almost perfect
Phytotherapeutics	2 (1.4)	0.43	Moderate

#### Agreement between observers

Table 3 shows the percentage of absolute agreement between the eight pharmaceutical observers according to ATC group (*n* = 140 drugs). There was substantial or almost perfect interobserver agreement for the majority of the drug classes in the guide, such as the main anatomical groups H, L, M, R, and S as well as the main therapeutic groups A02, C05, C07, C08, G03, and N03. However, there was only fair or slight interobserver agreement for antidiarrheals, intestinal anti-inflammatory/anti-infective agents, antithrombotic agents, and other dermatological preparations.

#### Agreement between each observer and the expert pharmacist

Table 4 shows the agreement between each observer and the gold standard. We obtained an overall kappa value of 0.78 [95% CI (0.66, 0.89)], which indicated almost perfect concordance between the observers and the expert pharmacist, and the overall agreement was 89.30% for the 140 drugs.

**Table 4:** Concordance between the eight observers and the expert pharmacist

Observer	Kappa value	SE	*p*	95% CI	Agreement
Observer 1	0.82	0054	< 0.001	0.71–0.92	Almost perfect
Observer 2	0.83	0050	< 0.001	0.73–0.93	Almost perfect
Observer 3	0.75	0059	< 0.001	0.64–0.87	Substantial
Observer 4	0.77	0060	< 0.001	0.65–0.88	Substantial
Observer 5	0.79	0057	< 0.001	0.68–0.90	Substantial
Observer 6	0.81	0054	< 0.001	0.70–0.91	Almost perfect
Observer 7	0.74	0062	< 0.001	0.62–0.86	Substantial
Observer 8	0.75	0061	< 0.001	0.63–0.87	Substantial

*SE* standard error, *95% CI* confidence interval

## Discussion

In this study, we developed a valid guide for the continuity of care in perioperative medication management, based on the available evidence and approved by a committee including specialists and physicians from the Department of Anesthesiology. This guide was validated by demonstrating that its use resulted in high concordance among eight pharmacists in decisions made regarding 140 drugs taken by 20 chronic inpatients. For the set of pharmaceutical interventions considered by the eight observers, we obtained an overall agreement of 86.9% and a kappa value of 0.7. When we compared the decisions made by the individual observers to those made by the expert pharmacist, the kappa value (a measure of the agreement between two observers) increased to 0.8 [95% CI (0.7, 0.9)], indicating almost perfect concordance, and the overall agreement was 89.3%.

A similar study was conducted in a tertiary hospital in Australia by Lindsay et al. [18] during 2013, where the aims were to design and validate deprescribing guidelines for cancer patients in palliative care and to identify potentially inappropriate medicines. That prospective, noninterventional cohort study comprised four major stages (similar to our study): developing the OncPal Deprescribing Guidelines based on current evidence; the prospective recruitment of consecutive palliative cancer inpatients; the assessment of all medications by a panel of medical experts to identify potentially inappropriate medicines; and an evaluation of the guidelines by concordance testing. The OncPal Deprescribing Guidelines matched 94.0% of the expert panel’s recommendations for 617 medicines, and the kappa value was 0.8 [95% CI (0.8, 0.9)], a similar result to ours. However, the difference from our study was that the Australian observers did not receive a training session regarding the guidelines because they were considered experts. In our study, we included pharmacists with a range of expertise in the evaluations, so the training course was crucial to achieving these great results. However, although concordance was very high for the majority of the medicine classes, it was low for antidiarrheals, intestinal anti-inflammatory/anti-infective agents, antithrombotic agents, and other dermatological preparations. When interpreting our results, it is important to note that the observers had no previous experience with this analysis, and that they carried out the observations that form the basis of this study only after a period of formal training. We feel that with additional experience, the results would have been better in all the drug classes.

It is important to note that his study has various limitations:

- This study focused only on the diagnosis of each patient undergoing an orthopedic procedure. Thus, previous comorbidities could have affected patient health.
- This study should have considered patients from different ethnic groups, given that the pharmacokinetics and pharmacodynamics of drugs can vary among ethnic groups [20].
- This study was carried out in just one hospital; in the future, the guide should be validated further and its reproducibility should be checked by applying it in different clinical settings in the same hospital and in different hospitals.

In summary, we have developed a guide for the continuity of care in perioperative medication management as a tool to improve the rationalization of medicines in the perioperative environment. Given the high number of medical comorbidities suffered by the elderly, and the associated polypharmacy and perioperative risks, it is important to ensure optimal management of the pre-existing medical conditions of these patients before and during surgery. Applying the guide developed here minimizes chronic disease progression or decompensation, interactions with anesthesia, and perioperative complications. The validation of this guide showed a high level of concordance between the trained observers and the expert who had previously classified the medication. Formal training seems to be essential to assure consistency of medication management, even among pharmacists with different levels of expertise.
